# Human *V_H_1-69* Gene-Encoded Human Monoclonal Antibodies against Staphylococcus aureus IsdB Use at Least Three Distinct Modes of Binding To Inhibit Bacterial Growth and Pathogenesis

**DOI:** 10.1128/mBio.02473-19

**Published:** 2019-10-22

**Authors:** Monique R. Bennett, Jinhui Dong, Robin G. Bombardi, Cinque Soto, Helen M. Parrington, Rachel S. Nargi, Clara T. Schoeder, Marcus B. Nagel, Kevin L. Schey, Jens Meiler, Eric P. Skaar, James E. Crowe

**Affiliations:** a Department of Pathology, Microbiology and Immunology, Vanderbilt University Medical Center, Nashville, Tennessee, USA; b Vanderbilt Vaccine Center, Vanderbilt University Medical Center, Nashville, Tennessee, USA; c Department of Chemistry, Vanderbilt University, Nashville, Tennessee, USA; d Department of Biochemistry, Vanderbilt University, Nashville, Tennessee, USA; e Department of Pediatrics, Vanderbilt University School of Medicine, Nashville, Tennessee, USA; f Vanderbilt Institute for Infection, Immunology and Inflammation, Vanderbilt University Medical Center, Nashville, Tennessee, USA; GSK Vaccines

**Keywords:** *Staphylococcus aureus*, X-ray crystallography, adaptive immunity, antibody functions, antibody repertoire, computer modeling, humoral immunity, monoclonal antibodies, proteomics

## Abstract

Staphylococcus aureus is an important human pathogen that infects nearly every human tissue. Like most organisms, the acquisition of nutrient iron is necessary for its survival. One route by which it obtains this metal is through the iron-regulated surface determinant (Isd) system that scavenges iron from the hemoglobin of the host. We show that the heavy chain variable region *IGHV1-69* gene commonly encodes human monoclonal antibodies (mAbs) targeting IsdB-NEAT2. Remarkably, these antibodies bind to multiple antigenic sites. One class of *IGHV1-69*-encoded mAbs blocks S. aureus heme acquisition by binding to the heme-binding site of NEAT2, while two additional classes reduce the bacterial burden *in vivo* by an alternative Fc receptor-mediated mechanism. We further identified clonal lineages of *IGHV1-69*-encoded mAbs using donor samples, showing that each lineage diversifies during infection by somatic hypermutation. These studies reveal that *IGHV1-69-*encoded antibodies contribute to a protective immune response, furthering our understanding of the correlates of protection against S. aureus infection.

## Abstract (precis)

The human pathogen Staphylococcus aureus causes a wide range of infections, including skin abscesses and sepsis. There is currently no licensed vaccine to prevent S. aureus infection, and its treatment has become increasingly difficult due to antibiotic resistance. One potential way to inhibit S. aureus pathogenesis is to prevent iron acquisition. The iron-regulated surface determinant (Isd) system has evolved in S. aureus to acquire hemoglobin from the human host as a source of heme-iron. In this study, we investigated the molecular and structural basis for antibody-mediated correlates against a member of the Isd system, IsdB. The association of immunoglobulin heavy chain variable region *IGHV1-69* gene-encoded human monoclonal antibodies with the response against S. aureus IsdB is described using structural and functional studies to define the importance of this antibody class. We also determine that somatic hypermutation in the development of these antibodies hinders rather than fine-tunes the immune response to IsdB.

## INTRODUCTION

Staphylococcus aureus is a Gram-positive pathogen that can cause illnesses ranging from skin and soft tissue infection to sepsis. The importance of understanding S. aureus pathogenesis is compounded because this pathogen persistently colonizes 20 to 50% of the population on the skin or in the nares (1). Because S. aureus is one of the leading causes of morbidity and mortality as well as a significant financial burden to the health care system (1), there is considerable effort invested into developing a vaccine or alternative immunotherapy drug. To be successful, the immune factors that protect individuals from initial or reoccurring S. aureus infection (2) need to be reliably identified and defined. To address this, we investigated the molecular and structural basis for antibody-mediated correlates against a member of the Isd system that is a known antibody target, IsdB (3–6). IsdB is part of the S. aureus iron-regulated surface determinant (Isd) system, which plays an important role in the acquisition of iron from the human host (7, 8). Almost all organisms, including S. aureus, need iron, as it serves as a cofactor for enzymes in metabolism and redox (9). The Isd system enables S. aureus to capture hemoproteins at the bacterial surface through two receptors, IsdB and IsdH (10, 11). IsdB removes heme from hemoglobin and transports it to downstream Isd proteins, where heme is transported into the cell and degraded so that iron is released for use in fundamental cellular processes (12). IsdB binds hemoglobin and heme using widely conserved NEAr iron transport (NEAT) domains (13, 14). IsdB has two NEAT domains: NEAT1 binds to hemoglobin, and NEAT2 is important for the transport and binding of heme (11, 15–17).

Some antibody repertoire studies have shown an association of certain human antibody variable genes with antigen-specific responses, such as the use of IGHV1*02 for HIV-specific VRC01-like antibodies to human CD4 (18) or certain *IGHV1-69* alleles and influenza virus hemagglutinin (HA) stem epitopes (19–22). *IGHV1-69* is one of the most common antibody variable genes with target-specific associations, as it encodes a hydrophobic heavy chain complementarity-determining region 2 (CDR-H2) motif that is optimal for engaging many recessed hydrophobic pocket regions (23). It has been observed previously for S. aureus that *IGHV1-69* encodes antibodies that preferentially bind to IsdB-NEAT2 (24), due to the hydrophobic CDR-H2 encoding a loop that has the capacity to bind to the heme-binding site on IsdB-NEAT2. Here, we show that *IGHV1-69* encodes not only IsdB-NEAT2 heme-binding-site antibodies but also antibodies that bind to at least two other antigenic sites on IsdB-NEAT2, likely using a heavy chain complementarity-determining region 3 (CDR-H3)-dominant mode of binding that does not depend critically on the hydrophobic nature of the encoded CDR-H2. Aside from their unique structural features, these alternate classes of *IGHV1-69*-encoded antibodies exhibited functional differences in inhibition of S. aureus growth, binding, kinetics, and pathogenesis *in vivo*. Deep sequencing of antibody gene repertoires revealed robust diversification of *IGHV1-69*-encoded antibody lineages by somatic hypermutation.

## RESULTS

### Structures of three *IGHV1-69*-encoded antibodies reveal three different modes of binding to NEAT2.

We previously described the isolation of a large panel of human monoclonal antibodies (mAbs) specific for binding to the NEAT2 domain of IsdB (IsdB-NEAT2) from two human subjects (5). Seven of the antibodies were encoded by the *IGHV1-69* antibody variable gene, which encodes hydrophobic residues in CDR-H2 in the germ line configuration. To determine if all these antibodies used the canonical hydrophobic PIF loop (amino acid positions 52 to 54) to interact directly with the heme-binding pocket on NEAT2, we determined the antigen-antibody complex structures for three representative *IGHV1-69*-encoded NEAT2-specific mAbs and compared their modes of interaction. We determined the crystal structure of mAb STAU-281 in complex with NEAT2, and for the other two mAbs, we used a hybrid structural method incorporating Fab crystallography, epitope mapping via hydrogen-deuterium exchange mass spectrometry (HDX-MS), and Rosetta modeling to obtain equivalent antigen-antibody complex structures (25, 26).

**FIG 1:**
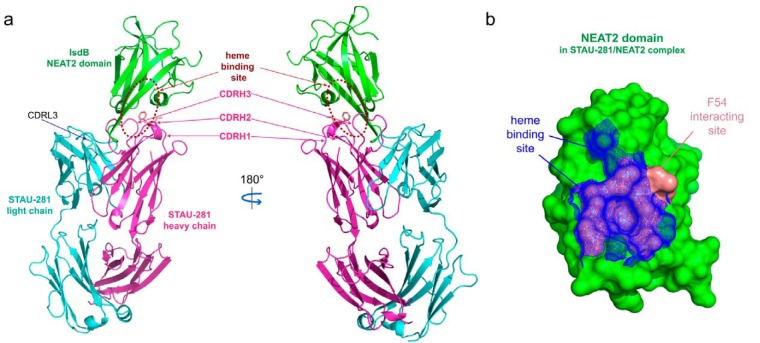
Crystal structure of STAU-281 Fab in complex with the IsdB-NEAT2 domain. (a) The STAU-281 heavy chain and light chain and the IsdB NEAT2 domain are shown in magenta, cyan, and green, respectively. The heme-binding site of the IsdB-NEAT2 domain is marked with broken ovals. Side chains of residues I53 and F54 (Kabat numbering) from CDR-H2 are shown in salmon in a stick representation. (b) Surface representation (green) of the NEAT2 domain in the STAU-281/NEAT2 complex crystal structure. The STAU-281 CDR-H2 residue F54 interaction site of the NEAT2 domain is in salmon. Equivalent atoms of the heme-binding site in the heme-bound NEAT2 structure (PDB accession number 3RTL) are mapped onto the NEAT2 domain in the STAU-281/NEAT complex structure as blue mesh.

We determined the crystal structure of human antibody STAU-281 Fab in complex with the NEAT2 domain of IsdB (Protein Data Bank [PDB] accession number 6P9H). The complex was crystallized in the space group of P21 with a diffraction limit of 3.00 Å (see Table S1 in the supplemental material), and there are two copies of the complexed structures in the asymmetric unit. The two copies of the complexed structures are superimposable, with a root mean square distance (RMSD) of 0.345 Å for C_α_ atoms. Complex formation buries an ∼1,011-Å^2^ surface area, and the heavy chain makes up more than 80% of the antigen-antibody interface (Fig. 1a). The search models used for the STAU-281 Fab-IsdB complex were the NEAT2 structure under PDB accession number 5D1X and the Fab under PDB accession number 5JRP.

10.1128/mBio.02473-19.8 TABLE S1 Data collection and refinement statistics for the crystals. Download Table S1, PDF file, 0.1 MB. Copyright © 2019 Bennett et al. 2019 Bennett et al. This content is distributed under the terms of the Creative Commons Attribution 4.0 International license.

**FIG 2:**
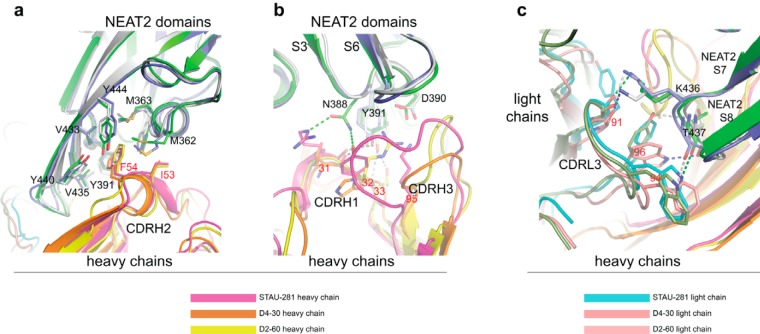
Overlay of crystal structures of STAU-281, D4-30 (PDB accession number 5D1X), and D2-06 (PDB accession number 5D1Q) Fabs in complex with the IsdB-NEAT2 domain. For the STAU-281/NEAT2 complex, the NEAT2 domain is in green. For the D4-30/NEAT2 complex, NEAT2 is in light blue. For the D2-06/NEAT2 complex, NEAT2 is in gray. (a) Residues I53 and F54 (Kabat numbering) of the conserved sequence motif of CDR-H2 encoded by the heavy chain gene *IGHV1-69* interact with the heme-binding site of the NEAT2 domain, including residues M362, M363, Y391, V435, Y440, V433, and Y444, via π-π stacking and hydrophobic interactions. (b) Variations in interactions between CDR-H1/CDR-H3 and NEAT2 S3/S4 and S5/S6 loops shown in the three complex structures. In the D4-30/NEAT2 complex, a main-chain oxygen at position 31 of D4-30 CDR-H1 forms an H bond with the NEAT2 S3/S4 loop Y391 side chain, and its CDR-H3 has little interaction with NEAT2. In the D2-60/NEAT2 complex, Y391 forms two H bonds with the main-chain nitrogen and the side chain of T33 of D2-60 CDR-H1, and there is a salt bridge between S3/S4 loop D390 and R32 of D2-60 CDR-H1. CDR-H3 of D2-60 also interacts extensively with NEAT2 loops. In the STAU-281/NEAT2 complex, besides the H bond between side chain Y391 and the main-chain oxygen of R31 at STAU-281 CDR-H1, two additional H bonds are found between the NEAT2 residue N388 side chain and the R31 side chain and the Y32 side chain of STAU-281 CDR-H1. A salt bridge is formed between NEAT2 D390 and STAU-281 CDR-H3 K95 instead of a residue from CDR-H1. Due to its longer CDR-H3, STAU-281 has extensive van der Waals interactions with the NEAT2 S5/S6 loop. (c) Shape complementarity between the NEAT2 domain S7/S8 loop and CDR-L3 of the kappa light chain of the three antibodies. Aromatic residues at positions 94 and 96 of CDR-L3 interact with the S7/S8 loop via van der Waals interactions in addition to hydrogen bonds (shown as broken lines). STAU-281/NEAT2 H bonds are in green, D4-30/NEAT2 H bonds are in gray, and D2-60/NEAT2 H bonds are in blue. The side chain of K436 of NEAT2 forms H bonds with main-chain oxygen atoms of CDR-L3 position 91 in all three complexes.

STAU-281 binds to the heme-binding pocket of NEAT2 and thus is expected to inhibit S. aureus growth by preventing heme from binding to IsdB (Fig. 1a). The structure revealed a major epitope, which we designate here IsdB antigenic site 1, where the hydrophobic CDR-H2 protrudes into the similarly hydrophobic heme-binding site on NEAT2. The footprint of the heme interaction with NEAT2 and the contact region of STAU-281 Phe-54 (Kabat numbering) of germ line-encoded CDR-H2 have highly overlapping binding sites (Fig. 1b), showing that this antibody inhibits S. aureus at least in part by blocking the acquisition of heme. We compared this crystal structure with those of two previously reported anti-NEAT2 antibodies (24), D2-06 (PDB accession number 5D1Q) and D4-30 (PDB accession number 5D1X) (Fig. 2). In general, the antigenic site recognized by STAU-281 was similar to those of D2-06 and D4-30. All three mAbs have a heavy chain encoded by the *IGHV1-69* variable gene and pair with a kappa light chain. The antibodies interact using three features: (i) the CDR-H2 loop, (ii) the CDR-H3 loop, and (iii) the light chain CDR-L3 loop. The binding of STAU-281 on NEAT2 differs in important details from those of the antibodies in the D2-06/NEAT2 and D4-30/NEAT2 complexes. The CDR-H2 interactions are nearly identical in all three complexes, while deviations were observed for the other loop interactions.

CDR-H2 of STAU-281 includes the sequence motif PIF (residues Pro-52A, Ile-53, and Phe-54 [Kabat numbering]) encoded by the *IGHV1-69* germ line sequence (Fig. 1a and b and Fig. 2a). The CDR-H2 residue F54 side chain of STAU-281 is inserted into the heme-binding pocket of the NEAT2 domain, forming extensive aromatic stacking interactions and hydrophobic interactions with surrounding aromatic and hydrophobic residues (valine and methionine residues) from the NEAT2 domain. The adjacent CDR-H2 residue I53 interacts with residues M362 and F366 of the NEAT2 heme-binding pocket (Fig. 2a). The residues in the NEAT2 domain interacting with CDR-H3 residues I53 and F54 overlap those of the NEAT2 heme-binding site (Fig. 1b).

In contrast to the stereotypical interaction of the CDR-H2s of the three heme-blocking antibodies described above, more variation was observed for the contact region and orientation of the CDR-H3 and CDR-H1 loops of STAU-281 and the two previously reported antibodies (Fig. 2b). In the STAU-281/NEAT2 and D4-30/NEAT2 complexes, the NEAT2 residue Y391 side chain forms an H bond with the CDR-H1 residue T31 main-chain oxygen, while in the D2-60/NEAT2 complex, the Y391 side chain forms H bonds with the side chains of T33 from CDR-H1 and D95 from CDR-H3. There is a salt bridge between NEAT2 D390 and STAU-281 CDR-H3 K95. In the D2-60/NEAT2 complex, residue R32 in CDR-H1, instead of residues from CDR-H3, forms a salt bridge with NEAT2 D390. However, the D4-30/NEAT2 complex lacks this cognate salt bridge. In the STAU-281/NEAT2 complex, NEAT2 residue N388 forms H bonds with STAU-281 CDR-H1 R31 and Y32 side chains. Moreover, STAU-281 has a longer CDR-H3, and CDR-H3 has extensive van der Waals interactions with NEAT2 loops. There is no contact between CDR-H3 and NEAT2 loops in the D4-30/NEAT2 complex, while CDR-H3 of D2-60 makes contacts with NEAT2 loops different from those in the STAU-281/NEAT2 complex.

The biased pairing of the kappa light chain with an *IGHV1-69*-encoded heavy chain in all three of these heme-blocking antibodies can be explained in part by the shape complementarity between the elongated β-turn of strand S7/S8 in the NEAT2 domain and the CDR-L3s of these kappa chains (Fig. 2c). In addition, there are two H bonds between the β-turns and the CDR-L3s. In the STAU-281/NEAT2 complex, an H bond forms between the W94 side chain NE1 atom in CDR-L3 and the T437 main-chain oxygen, and another bond forms between the Y91 main-chain oxygen in CDR-L3 and the K436 side chain in NEAT2.

### Recognition of alternate antigenic sites by *IGHV1-69*-encoded antibodies.

We next sought to determine the antigenic sites recognized by STAU-399 and STAU-229. Despite their common *IGHV1-69* germ line usage and protein target, we found that mAbs STAU-399 and STAU-229 bind to IsdB-NEAT2 at epitopes distal from the heme-binding site and rely mostly on CDR-H3 (specified by diverse V_H_-N1-D_H_-N2-J_H_ junctions formed during VDJ recombination) as the principal interacting antibody loop, instead of the *IGHV1-69* germ line gene-encoded hydrophobic CDR-H2. We generated high-resolution models of the interactions using crystallography and HDX-MS.

Efforts to crystallize NEAT2 in complex with STAU-399 or STAU-229 were unsuccessful. We were, however, successful in obtaining crystal structures of Fab fragments for the mAbs STAU-399 (2.4-Å resolution) (PDB accession number 6P9I) and STAU-229 (2.1-Å resolution) (PDB accession number 6P9J). The search model used was the Fab under PDB accession number 5JRP. Both STAU-399 and STAU-229 contain longer CDR-H3 loops than those of STAU-281. We also noted that the CDR-L3 loop of STAU-281, which sits opposite the binding cavity on NEAT2, is shorter for STAU-281 than for STAU-399 or STAU-229, leaving more space for the engagement of the NEAT2 domain. Taken together, the structures suggest that in heme-blocking *IGHV1-69*-encoded antibodies, the hydrophobic patch in the *IGHV1-69*-encoded CDR-H2 should be combined with a shorter CDR-H3 loop and a light chain that can accommodate the tip of the domain to bind the NEAT2 domain at the heme-binding pocket. The STAU-399 and STAU-229 antibodies fail to satisfy these structural requirements.

**FIG 3:**
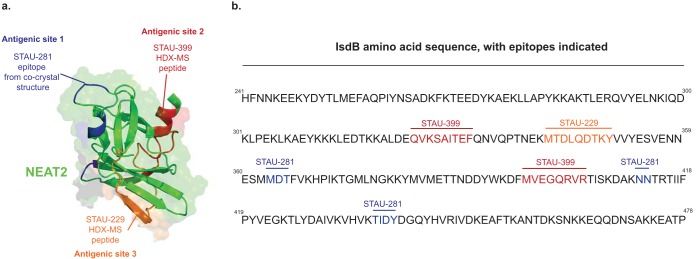
Epitopes for two *IGHV1-69*-encoded antibodies on NEAT2. Hydrogen-deuterium exchange mass spectrometry (HDX-MS) was used to map the binding sites of STAU-229 (orange) or STAU-399 (red). (a) Peptides participating in the two epitopes were determined by reduced labeling in the presence of antibody and visualized on the surface of the IsdB-NEAT2 domain (PDB accession number 3RTL), shown in yellow ribbon and surface projection. The binding site of STAU-281 as determined by X-ray crystallography of a complex of STAU-281 Fab and recombinant NEAT2 is indicated in blue for reference. (b) Peptides in epitopes for STAU-229 (orange) or STAU-399 (red) in the amino acid sequence of IsdB and contact residues in the cocrystal structure for STAU-281/NEAT2 (blue).

To determine the binding sites for STAU-399 and STAU-229 using HDX-MS, we identified peptides on the surface of NEAT2 recognized by STAU-399 (Fig. 3 and Fig. S1) or STAU-229 (Fig. 3 and Fig. S2). The reduction of deuterium exchange in the presence of Fab suggested NEAT2 peptides that interact with Fab. For STAU-399, we found that labeling of the NEAT2 peptides QVKSAITEF (residues 324 to 332) and MVEGQRVR (residues 396 to 403) reduced deuterium incorporation in the presence of Fab. We projected this epitope onto the surface of NEAT2 and designated this region antigenic site 2. For STAU-229, we found that the NEAT2 peptide MTDLQDTKY (residues 342 to 350) showed reduced deuterium incorporation in the presence of Fab. We projected this epitope onto the surface of NEAT2 and designated this region antigenic site 3.

10.1128/mBio.02473-19.2 FIG S1 HDX-MS of IsdB-NEAT with STAU-399. (a) Heat map showing the difference in relative deuterium uptake of IsdB in complex with STAU-399 Fab compared to that of unbound IsdB. The sequence of IsdB is shown, and overlapping peptides are shown at the bottom. Shielded amino acids (blue) indicate potential binding sites and are boxed. (b) Structure of crystallized IsdB. Coloring represents the difference in deuterium uptake in a complex compared to that of solitary IsdB from HDX. The scale ranges from −20 (blue) to +20 (red); black represents unobserved regions. Download FIG S1, PDF file, 0.5 MB. Copyright © 2019 Bennett et al. 2019 Bennett et al. This content is distributed under the terms of the Creative Commons Attribution 4.0 International license.

10.1128/mBio.02473-19.3 FIG S2 HDX-MS of IsdB-NEAT with STAU-229. (a) Heat map showing the difference in relative deuterium uptake of IsdB in complex with STAU-229 Fab compared to that of unbound IsdB. The sequence of IsdB is shown, and overlapping peptides are shown at the bottom. Shielded amino acids (blue) indicate potential binding sites and are boxed. (b) Structure of crystallized IsdB. Coloring represents the difference in deuterium uptake in a complex compared to that of solitary IsdB from HDX. The scale ranges from −20 (blue) to +20 (red); black represents unobserved regions. Download FIG S2, PDF file, 0.7 MB. Copyright © 2019 Bennett et al. 2019 Bennett et al. This content is distributed under the terms of the Creative Commons Attribution 4.0 International license.

**FIG 4:**
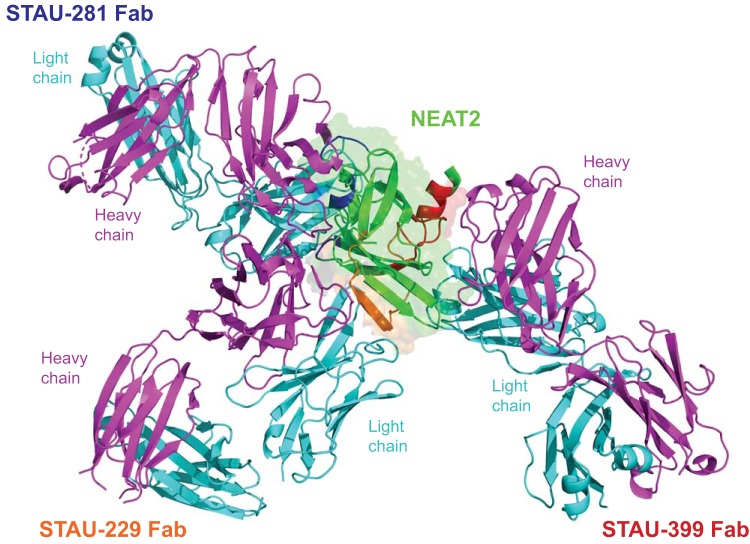
Overlay model of the structures of three *IGHV1-69*-encoded antibodies reveals three modes of binding to NEAT2. The structures of the *apo* forms of Fabs STAU-229 (2.1 Å) and STAU-399 (2.4 Å) were determined by crystallography. These two Fabs were docked onto the STAU-281/NEAT2 complex structure using the Rosetta modeling suite, restricting docking to their binding sites (orange and red, respectively) predicted by HDX-MS studies of NEAT2 and the Fabs (Fig. 2). Heavy chains are shown as cyan ribbons, and light chains are shown in green.

Next, we built high-resolution models of the antigen-antibody complexes using a hybrid-method approach by combining the information from the experimental Fab structures and from the HDX-MS experiments. The interaction of Fab and antigen for each antibody was determined by docking the *apo* Fab and *apo* IsdB crystal structures (PDB accession number 5VMM) (17) using the Rosetta modeling software suite (25), with restrictions to the HDX-MS data (Fig. 4). These experiments revealed that the three antibodies bind to NEAT2 in very distinct sites and poses. The interaction with antigenic site 2 (defined by STAU-399 binding) showed that the long CDR-H3 of STAU-399 plays a key role in the recognition of this site, interacting near α-helix 3 of NEAT2 (Fig. 4). The canonical F54 residue on CDR-H2 of STAU-399 still interacts with IsdB but is removed from the heme-binding site. The interaction of STAU-299 with antigenic site 3 shows recognition of Isd-NEAT2 near α-helix 3, using STAU-299 CDR-H3 as the predominant interacting loop (Fig. 4).

### *In vitro* blocking of heme binding mediated by *IGHV1-69*-encoded mAbs.

**FIG 5:**
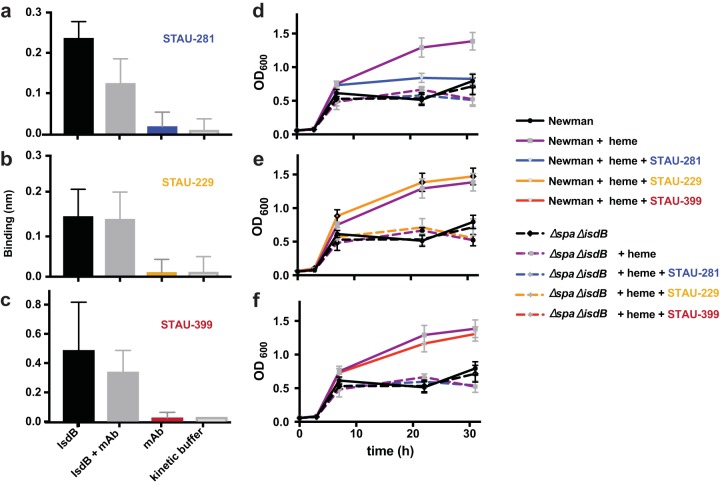
*In vitro* blocking of binding (a to c) or inhibition of growth of S. aureus (d to f) mediated by *IGHV1-69*-encoded mAbs. (a to c) Biolayer interferometry was used to detect whether the mAbs blocked binding of hemoglobin to NEAT2. Biotinylated hemoglobin was loaded onto streptavidin-coated biosensor tips before association with either IsdB alone, IsdB mixed with mAb, mAb alone, or kinetic buffer. Error bars represent standard deviations. *P* values were determined by an unpaired *t* test. This experiment was performed at least 3 independent times. (d to f) S. aureus heme-dependent *in vitro* growth curves were performed in 96-well plates over 32 h. S. aureus strain Newman was subcultured at a 1:200 dilution into RPMI medium with ethylenediamine-*N*,*N*′-bis(2-hydroxyphenylacetic acid) and normalized to an OD_600_ of 1.0. Error bars represent standard deviations. Data shown are representative of results from 3 independent experiments.

The biological significance of the antigenic sites for blocking the interaction of heme or hemoglobin with IsdB was tested using two separate *in vitro* assays. First, each *IGHV1-69*-encoded mAb was tested for its ability to block heme or hemoglobin binding to IsdB using a biolayer interferometry biosensor. Biotinylated hemoglobin was loaded onto streptavidin sensors and then associated with IsdB alone, mAb alone, IsdB plus mAb, or buffer (Fig. 5a to c). The only antibody that significantly blocked the IsdB-hemoglobin binding interaction was STAU-281. Conversely, when STAU-229 and STAU-399 were incubated with IsdB and associated with hemoglobin, we did not detect a difference in the binding signal compared to those for IsdB and hemoglobin binding alone. STAU-281 not only directly binds to the heme-binding pocket but also prevents binding of hemoglobin to IsdB. This mode of action is similar to that of the previously described mAbs D2-06-N2 and D4-30-N2 (24).

### *In vitro* inhibition of growth mediated by *IGHV1-69*-encoded mAbs.

Next, the mAbs were tested for their ability to inhibit *in vitro* replication of S. aureus when grown in the presence of heme as a sole iron source. S. aureus strain Newman (wild type [WT]) or the protein A- and *isdB*-deficient Δ*spa* Δ*isdB* strain was grown overnight and then subcultured into 96-well plates containing RPMI medium plus ethylenediamine-*N*,*N′*-bis(2-hydroxyphenylacetic acid) (EDDHA) to reduce the amount of available iron. When heme was added to wells containing the Δ*spa* Δ*isdB* strain, there was no increase in growth due to a lack of functional *isdB* (11). In contrast, the addition of heme led to an increase in the growth of wild-type S. aureus to an optical density at 600 nm (OD_600_) of ∼1.5 (Fig. 5d to f). STAU-281 reduced growth when added to the medium, while an inhibitory effect was not detected for the STAU-399- or STAU-229-treated groups, in which the OD values did not differ from those of the group treated with heme alone. These data show that representative mAbs that bind to antigenic site 2 or 3 on NEAT2 do not prevent heme or hemoglobin binding and do not inhibit wild-type strain growth using heme as a sole iron source *in vitro*, in contrast to STAU-281, which inhibits S. aureus growth.

### All three classes of *IGHV1-69*-encoded mAbs reduce bacterial burdens during infection.

Next, these mAbs were tested in a murine septic model of S. aureus infection to determine whether mAb binding to any of the three major antigenic sites on NEAT2 mediates a protective effect *in vivo*. Two variant forms of *IGHV1-69*-encoded mAbs were tested in this infection model, full-length IgG1 with a wild-type Fc region or IgG1 Fc variant antibodies with L234A/L235A (LALA) mutations in the CH2 domain, which reduces antibody binding to FcγR receptors (27, 28). The interaction between FcγR receptors and the Fc portion of an antibody mediates secondary functions such as phagocytosis and antibody-dependent cytotoxicity. Therefore, mutating these critical residues can reveal whether Fc effector functions are an important part of the inhibitory effect of antibodies *in vivo* (29).

**FIG 6:**
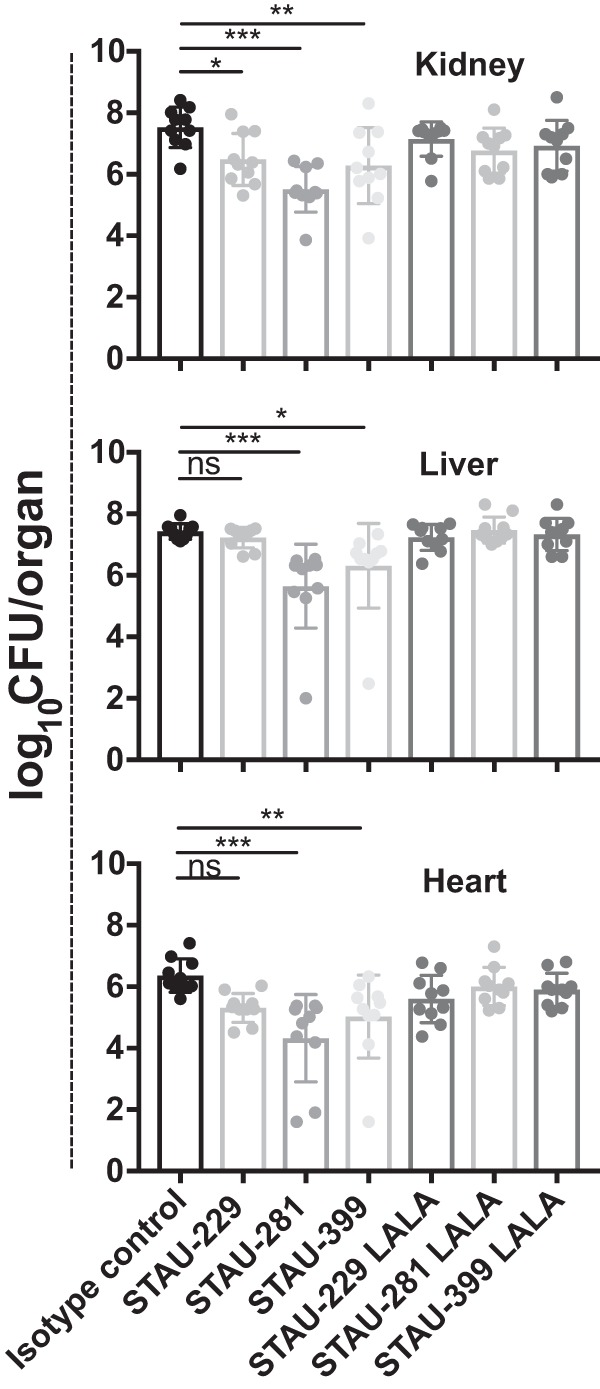
*IGHV1-69*-encoded mAbs reduce bacterial burden *in vivo.* Seven-week-old female BALB/c mice were inoculated retro-orbitally with a suspension of S. aureus strain Newman at an OD_600_ of 0.4. Mice were given *IGHV1-69*-encoded full-length wild-type IgG LALA Fc variant (L234A/L235A) IgG antibodies by the intraperitoneal route. The hearts, livers, and kidneys of the infected mice were harvested after 96 h. Statistical significance was evaluated by analysis of variance (ANOVA) using multiple comparisons. All other comparisons, including comparison of LALA mAbs to the isotype control, were not significant. Experiments were performed two independent times, and the pooled data are shown. *P* values of <0.0001 were considered significant. *, *P* < 0.05; **, *P* < 0.01; ***, *P* < 0.001; ns, not significant.

Seven-week-old BALB/c mice were given one of three *IGHV1-69*-encoded mAbs by intraperitoneal injection and then inoculated retro-orbitally with 10^7^ CFU of wild-type S. aureus. Tissues were harvested after 96 h, and the bacterial burden was enumerated by serial dilution plating (Fig. 6). Treatment with the IgG1 form of mAb STAU-281 caused a significant reduction in the bacterial burden compared to those for isotype-control-treated animals in all three organs tested, including a >100-fold reduction in the bacterial burden in the heart and kidneys and a 60-fold reduction in the liver. While STAU-399 did not block binding of hemoglobin or heme *in vitro*, it significantly reduced the burden in the kidneys, liver, and heart. STAU-229 significantly reduced the burden in the kidneys. In contrast, we did not detect a significant reduction in bacterial burden in any tissue for any of the LALA mutant IgGs, compared to those in mice treated with isotype control IgG. Therefore, binding of the full-length *IGHV1-69*-encoded IgG mAbs to FcγR contributes to the protective mechanism of these mAbs.

### Antibody repertoire analysis reveals clonal lineages of *IGHV1-69*-encoded mAbs containing clones with diverse patterns of somatic mutations.

We next identified somatic variants of the mAbs to determine if clonal lineages developed and if affinity maturation of the clonotypes affected the function of the protective *IGHV1-69-*encoded antibodies. We used an additional aliquot of cryopreserved peripheral blood mononuclear cells (PBMCs) collected in the convalescent period from subject 924 and performed antibody heavy chain repertoire sequencing. We obtained 3,395,084 unique and productive heavy chain variable gene sequences from approximately 1.1 million PBMCs (estimated by taking 7% of the total number of PBMCs). To minimize the influence of potential sequencing errors, we employed the concept of a V3J clonotype, which uses the variable (V) and joining (J) germ line genes (ignoring allelic distinction) along with the CDR3-H3 amino acid sequence (30). The reduction of the total pool of unique and productive heavy chain reads according to the clonotype definition resulted in the identification of a total of 1,047,493 unique V3J clonotypes.

**FIG 7:**
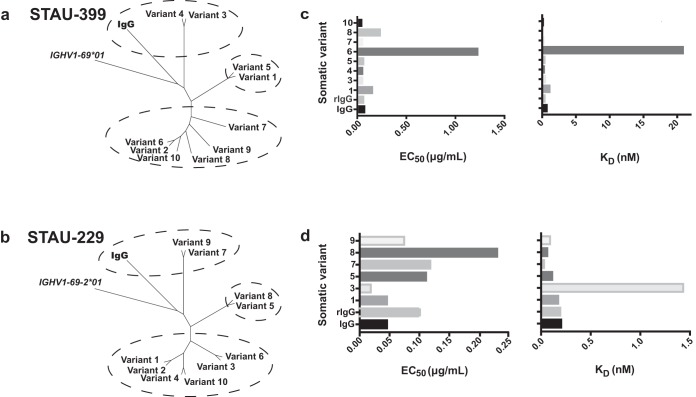
Repertoire analysis of somatic variants of *IGHV1-69*-encoded mAbs. Deep sequence analysis of antibody variable genes expressed in donor PBMCs revealed somatic variants for the heavy chains of STAU-399 and -229. These somatic variant heavy chain antibodies were expressed recombinantly with the original light chain and purified. (a and b) Neighbor-joining tree analysis of mAb clonal variants by Geneious. The germ line gene sequences of IGHV1-69*01 or IGHV1-69-2*01 were used as the outgroup sequences. (c and d) Each variant mAb was tested for binding to IsdB, and the half-maximal effective concentration (EC_50_) for binding was determined. Biolayer interferometry was used to determine the on and off rates of each variant antibody for binding to NEAT2, and the calculated *K_D_* is graphed. These experiments were performed three times independently.

Because STAU-399 and STAU-229 were isolated from the same donor for which we performed deep-sequencing analysis, we were able to search for somatic variants that were also *IGHV1-69-*encoded antibody sequences in that donor. The phylogenies of the somatic variants associated with the clonotypes from repertoire sequencing were then analyzed to infer a possible maturation pathway (Fig. 7a and b). Point mutations were identified in the V_H_ region, and we synthesized and tested these variant antibodies for differences in binding to IsdB (Fig. 7c and d).

Ten sequences of the STAU-399 clonotype (encoded by *V_H_1-69/J_H_6*) were identified, with 3 of them (variants 2, 8, and 9) being identical. Somatic variants within a lineage (“siblings”) clustered into 3 groups (Fig. 7a). STAU-399 IgG and variants 3 and 4 grouped together in cluster 1, based on their conservation of the germ line gene sequence encoding the CDR-H2 loop, which has a threonine at position 56 (Fig. S3). This finding is in contrast to those for all other variants that shared a T56A mutation in the CDR-H2 loop. Threonine likely participates in important hydrogen bonds with surrounding side chains and water, and the T56A mutation may alter the CDR-H2 loop interactions, making them more hydrophobic. The largest cluster of this clonotype is cluster 3, encoding 6 variants. Most of the mutations in this cluster did not lead to noticeable changes in the binding affinity of these variants, with the exception of variant 6, which has three mutations to glycine (V23G in framework region 1 [FR1], S30G in CDR1, and V36G in FR2), making this clone the most distant genetically from the germ line gene sequence. Correspondingly, this mAb exhibited the lowest affinity for binding (27-fold lower than for the hybridoma IgG) and the lowest 50% inhibitory concentration (EC_50_) value for binding in an enzyme-linked immunosorbent assay (ELISA) (18-fold lower than for the hybridoma IgG) (Fig. 7c). An additional IGHV1-69*01-encoded mAb, STAU-307, was found to have clonal variants within the donor sample and behaved similarly to STAU-399 (Fig. S4 and Fig. S5).

10.1128/mBio.02473-19.4 FIG S3 Sequence alignment of STAU-399 and clonal variants. A global alignment using Geneious was performed with the germ line IGHV1-69*01 sequence, hybridoma IgG, and all clonal variants. Each HCDR and framework (FR) region is denoted, with a consensus sequence shown. Point mutants that differ from the consensus are shown as individual amino acids, whereas conserved residues are represented as dots. Download FIG S3, PDF file, 0.3 MB. Copyright © 2019 Bennett et al. 2019 Bennett et al. This content is distributed under the terms of the Creative Commons Attribution 4.0 International license.

10.1128/mBio.02473-19.5 FIG S4 Neighbor-joining tree analysis and binding kinetics of STAU-307 and clonal variants. An additional IGHV1-69*01 antibody (which binds site 2), STAU-307, was found to have clonal variants within the donor sample. Ten sequences of the STAU-307 clonotype (encoded by V_H_1-69*01/J_H_4*02) were identified, with two of these sequences being identical. Siblings were divided into two main clusters based on the phylogenetic analysis. Cluster 1 contains the original IgG and variants 5 and 8 to 10, whereas cluster 2 contains variants 1 to 4, 6, and 7. Interestingly, the main differences between these two clusters occurs in the DE loop (see Fig. S2 in the supplemental material), where three different runs of mutations occur. All variants included in clusters 1 and 2 have mutations in their HCDR2 that differ from the germ line IGHV1-69*01 sequence. This finding is striking, as a conserved HCDR2 can have important implications for blocking the heme pocket of NEAT2, as Fig. 1 shows; however, none of the STAU-307 variants maintain 100% identity with the germ line sequence. Instead, all 10 variants and the sequence from hybridoma IgG have the mutations I51V and I53V. Although both isoleucine and valine are branched hydrophobic amino acids, mutation between these residues has been shown to alter function in previous studies (X. Yuan, P. Yin, Q. Hao, C. Yan et al., J Biol Chem, 285:28953–28958, 2010, doi:https://doi.org/10.1074/jbc.M110.160192, and J. T. Brosnan, M. E. Brosnan, J Nutr 136:1636S–1640S, 2006, doi:https://doi.org/10.1093/jn/136.6.1636S). Despite the presence of these point mutations, these variants had largely the same ability to bind IsdB when tested by an ELISA (Fig. 4E). Variant 1, however, had a noticeably lower *K_D_* than the other variants tested. This difference may be attributed to the Y107D mutation found in HCDR3 that is unique to this sequence. Download FIG S4, PDF file, 0.1 MB. Copyright © 2019 Bennett et al. 2019 Bennett et al. This content is distributed under the terms of the Creative Commons Attribution 4.0 International license.

10.1128/mBio.02473-19.6 FIG S5 Sequence alignment of STAU-307 and clonal variants. A global alignment using Geneious was performed with the germ line IGHV1-69*01 gene sequence, the sequence from the hybridoma encoding the original IgG, and all STAU-307 clonal variants. Each HCDR and FR is denoted, with a consensus sequence indicated. Point mutants that differ from the consensus are shown as individual amino acids, whereas conserved residues are represented as dots. Download FIG S5, PDF file, 0.3 MB. Copyright © 2019 Bennett et al. 2019 Bennett et al. This content is distributed under the terms of the Creative Commons Attribution 4.0 International license.

We identified nine sequences within the STAU-229 clonotype and three clusters of variants (encoded by V_H_1-69-2*01/J_H_6*02) with some common mutations noted (Fig. 7b and Fig. S6). Because STAU-229 is encoded by the IGHV1-69-2*01 allele, this mAb has a different inferred germ line gene sequence than that of STAU-399. The residues in positions 52 to 54, encoding the key amino acid motif PIF that was conserved in all of the IGHV1-69*01-encoded antibodies, are changed to PED. These alleles represent two distinct gene sequences. In the STAU-229 mAb heavy chain variable gene sequence and all 1,047,493 clonotype variants identified by repertoire sequencing, the PED motif was maintained. The I53E and F54D alternate residues encoded by the IGHV1-69-2*01 gene make this germ line-encoded CDR-H2 loop more polar and hydrophilic than that encoded by IGHV1-69*01. Interestingly, STAU-229 IgG is the only mAb isolated with a mutation in CDR-H2 (V50I), which is the same residue encoded by the germ line IGHV1-69*01, suggesting that these canonical hydrophobic isoleucine residues facilitate optimal binding to NEAT2. A number of STAU-229 variant sequences were shared within cluster 3, so we tested the representative sequences for variants 1 and 3 after recombinant expression. Variant 3 differs from variant 1 only by an S20P substitution in FR1; however, this difference was sufficient to alter the *K_D_* (equilibrium dissociation constant) of this antibody to 1.44 nM, a nearly 7-times-lower avidity than that of the hybridoma cell line-expressed IgG, making it the antibody with the worst *K_D_* with this clonotype (Fig. 7d).

10.1128/mBio.02473-19.7 FIG S6 Sequence alignment of STAU-229 and clonal variants. A global alignment using Geneious was performed with the germ line IGHV1-69-2*01 gene sequence, the sequence from the hybridoma encoding the original IgG, and all STAU-229 clonal variants. Each HCDR and FR is denoted, with a consensus sequence indicated. Point mutants that differ from the consensus are shown as individual amino acids, whereas conserved residues are represented as dots. Download FIG S6, PDF file, 0.3 MB. Copyright © 2019 Bennett et al. 2019 Bennett et al. This content is distributed under the terms of the Creative Commons Attribution 4.0 International license.

## DISCUSSION

S. aureus has proven to be a formidable pathogen for vaccine design. With multiple virulence strategies, redundant nutrient acquisition pathways, and a list of failed vaccines, it is critical to obtain a more thorough understanding of how to better target the relevant factors. A large body of work has shown that iron acquisition pathways are crucial for many living organisms to survive and that heme is necessary for full virulence in S. aureus. Moving forward, strategies to fully exploit this pathway for heme-iron acquisition may improve the efficiency and success of S. aureus vaccine design. This work not only contributes to a broader understanding of a biologically important antigen, IsdB, but also describes a panel of protective mAbs that exhibit therapeutic efficacy in S. aureus infection in mice.

In this study, we characterized three antigenic sites on IsdB-NEAT2 and their biological importance *in vitro* and *in vivo*. Although *IGHV1-69*-encoded antibodies bind principally by hydrophobic CDR-H2-mediated binding interactions (24), some antibodies to IsdB-NEAT2 do not use CDR-H2 as the primary binding loop. This observation is interesting, as key antibody-antigen interactions have been described for a number of pathogens using *IGHV1-69*-encoded antibodies, whether mediated by CDR-H2, CDR-H3, or both. For example, in influenza virus, the *IGHV1-69*-encoded antibody 27F3 broadly targets influenza group 1 and 2 viruses at the HA stem using the IFY motif on CDR-H2 (21). In hepatitis C virus (HCV), the *IGHV1-69*-encoded antibody AR3A-D broadly neutralizes HCV strains by binding to the E2 domain of HCV using a V_H_-encoded hydrophobic CDR-H2 and a long CDR-H3 formed during VDJ recombination (31). For HIV, investigators have identified *IGHV1-69*-encoded antibodies that predominantly use CDR-H2 or some that use both CDR-H2 and CDR-H3. mAb HK20 uses the traditional hydrophobic IF motif on CDR-H2 to target gp41 of HIV (32). In contrast, the *IGHV1-69*-encoded antibody VRC13 uses a CDR-H3-mediated mechanism to broadly neutralize HIV by recognizing and blocking gp120 function (33).

Genetic polymorphisms in *IGHV1-69* gene alleles could limit or affect the human antibody responses to the antigenic sites that were identified here. Thirteen *IGHV1-69* alleles have been described that possess F54L, T56I, and G49R polymorphisms, which are located in or near CDR-H2 (34). Seven alleles encode a phenylalanine at position 54 of the germ line gene sequence that mediates the canonical interaction with the heme-binding site, but six alleles encode a leucine at this position (L54). A substantial proportion of the general population is homozygous for alleles with L54, suggesting that these individuals may not be able to make *IGHV1-69*-encoded heme-blocking antibodies (35). Also, the number of *IGHV1-69* germ line copies per diploid human genome varies (35). If heme blocking of NEAT2 on IsdB is an important component of protective immunity, one might observe variability in the functional immune responses in recipients of experimental vaccine candidates due to the need to use a subset of *IGHV1-69* alleles. Similar to the studies that we describe here, repertoire studies with human antibody responses to the influenza virus hemagglutinin stem region show both canonical CDR-H2-mediated IGHV1-69*01 allele (F54 residue)-encoded interactions (36, 37) and noncanonical IGHV1-69*09 allele (non-F54)-encoded interactions mediated by CDR-H3 (38). It was interesting that the STAU-229 mAb described here is encoded by *IGHV1-69*-2*01, which lacks the F residue needed for heme-binding-site recognition, and indeed, this mAb recognizes an alternate antigenic site. Interactions with *IGHV1-69*-encoded antibodies are complex and depend on the allele, somatic mutations, the dominant interaction using CDR-H2 or CDR-H3, and the antigenic site recognized. Combinations or interactions of these antibodies could affect their performance *in vivo*. We tested STAU-281 (the heme-blocking mAb) in combination with multiple other antibodies, including *VH1-69*-encoded antibodies; however, the combinations that we tested did not lead to a reduction in bacterial burden greater than that with STAU-281 alone.

The discovery of diverse *IGHV1-69*-encoded antibodies that recognize distinct antigenic sites on an immunodominant gene target in S. aureus is intriguing. Identification of common germ line-encoded antibodies for a pathogen, as we have done here, represents a unique opportunity to use vaccination to shape a targeted antibody response. The increased understanding of structure-function aspects of *IGHV1-69-*encoded antibodies recognizing S. aureus that contribute to a protective immune response informs rational vaccine design efforts and enables a better understanding of the correlates of protection against S. aureus infection.

## MATERIALS AND METHODS

### Human subjects.

Human blood was collected from patients at Vanderbilt University Medical Center after written informed consent and subject assent. The studies were approved by the Institutional Review Board of Vanderbilt University Medical Center.

### Generation of human monoclonal antibodies.

Peripheral blood mononuclear cells (PBMCs) were isolated from deidentified blood samples by Ficoll density gradient centrifugation. Human hybridomas were generated by transforming PBMCs with medium containing Epstein-Barr virus, CpG (Life Technologies), a Chk2 inhibitor (Sigma), and cyclosporine (Sigma). Cells were expanded using a feeder layer of irradiated heterologous human PBMCs. Supernatants from expanded cells were screened by an ELISA using recombinant IsdB protein to identify wells containing B cells secreting antibodies reactive with IsdB. Transformed B cells in wells with a positive signal were fused with HMMA2.5 myeloma cells by electrofusion. Monoclonal hybridoma lines were obtained using single-cell sorting on a FACSAria III instrument.

### Bacterial strains.

S. aureus strain Newman was grown at 37°C for 12 to 18 h on tryptic soy agar plates or in tryptic soy broth. Isogenic knockouts were made in a Newman background by using allelic replacement of the coding sequence with *ermC* (7, 39, 40). PCR fragments were assembled into the pCR2.1 DNA plasmid vector and then recombined into the pKOR1 plasmid to inactivate the gene.

### Generation of recombinant IsdB and IsdB-NEAT2 proteins.

A cDNA of the sequence encoding IsdB, excluding the sorting signal and signal peptide, was cloned into the pET15b vector and expressed using Escherichia coli BL21(DE3) cells. For IsdB-NEAT2, only the NEAT2-encoding sequence was cloned into a pET15b vector. For both proteins, bacteria were grown in Luria-Bertani (LB) broth for 36 h total and induced after 6 h at 30°C with 1 mM isopropyl-β-d-thiogalactopyranoside (IPTG). The culture was harvested by centrifugation and resuspended in 50 mM Na_2_HPO_4_ plus 500 mM NaCl before disruption by homogenization (LM20 microfluidizer). The protein was purified using a HiTrap Talon column, and the His tag was cleaved with thrombin.

### Growth curves in the presence of heme.

S. aureus strain Newman or the Δ*isdB* Δ*spa* strain was grown overnight in RPMI medium containing 0.1 Casamino Acids and 0.5 mM EDDHA. The OD_600_ was normalized to a value of 1, and the bacteria were subcultured in 200 μl of RPMI medium plus EDDHA with 20 nM heme and 2 μg/ml mAb. Bacteria in 96-well plates were grown at 37°C, and OD_600_ values were recorded at 3, 7, 24, and 36 h using path-length correction in Gen5 microtiter plate software (Bio-Tek).

### Biolayer interferometry assays.

*K_D_* and blocking studies were performed using an Octet red biosensor (Pall FortéBio). For *K_D_* studies, individual antibodies were loaded onto anti-human Fc biosensors. Biosensor tips were first washed and then immersed into wells containing mAb (5 μg/ml), followed by an additional wash step, before association with wells containing 2-fold dilutions of IsdB protein. The initial IsdB concentration was converted from micrograms per milliliter to nanomolar units with a starting molarity of 150 nM. The *k*_on_ and *k*_off_ values for interactions were determined by global fitting of the curves in Octet software. Blocking studies were performed by first washing streptavidin biosensors and then associating them with biotinylated hemoglobin.

### Mouse experiments.

A murine septic model was used (5). Seven-week-old female BALB/cJ mice were injected with 10 mg/kg of body weight of mAb via the intraperitoneal route. Mice were anesthetized and injected retro-orbitally with 10^7^ CFU S. aureus strain Newman. After 96 h, mice were euthanized by CO_2_ inhalation, and organs were collected and homogenized in phosphate-buffered saline (PBS) before serial dilution for colony enumeration. Mouse experiments were approved and performed according to the guidelines of the Vanderbilt University School of Medicine Institutional Animal Care and Use Committee (IACUC).

### Hydrogen-deuterium exchange mass spectrometry.

IsdB and Fab proteins were prepared at 10 pmol/μl. Labeling occurred in PBS (pH 7.4) in D_2_O at 20°C for 30 s or 4 h. The reaction was quenched with a solution containing PBS, 4 M guanidinium-HCl, and 100 mM tris(2-carboxyethyl)phosphine to pH 2.3 at 0°C. Samples were injected into a nano-Acquity ultraperformance liquid chromatography (UPLC) system with HDX technology. Digestion was performed at 15°C with a flow rate of 134 μl/min of 0.1% formic acid using a pepsin column. Peptides were simultaneously trapped at 0°C on a VanGuard ethylene bridged hybrid (BEH) C_18_ 1.7-μm column. Peptides were separated on an Acquity UPLC BEH C_18_ 1.7-μm, 1-mm by 100-mm column; eluted using 5 to 35% acetonitrile and 0.1% formic acid in H_2_O; and analyzed using a Xevo G2-XS mass spectrometer in MS^E^ mode. Identification was performed using Waters ProteinLynx global server 3.0.3, and deuterium uptake was calculated using DynamX 3.0. Results were averaged across replicate analyses, at a given time point, and the standard deviation was determined.

### X-ray crystallography.

The IsdB-NEAT2 protein was run on size exclusion chromatography (SEC) columns (HiLoad 16/600, Superdex 75 pg; GE Healthcare Life Sciences) in buffer containing 20 mM Tris and 50 mM NaCl. The antigen was concentrated to 10 mg/ml and incubated at room temperature with Fab at a 1:2 ratio. The incubated complex was run on an SEC column and concentrated to 10 mg/ml. All crystals were obtained using Hampton Research screens under various conditions. X-ray diffraction data were collected at Advanced Photon Source LS-CAT beamline 21-ID-G or -F. STAU-281 in complex with IsdB-NEAT2 was crystallized in a solution containing 0.1 M sodium citrate tribasic dehydrate (pH 5.5) and 16% polyethylene glycol 8000 (PEG 8000). STAU-399 Fab was crystallized in a solution containing 10% PEG 200, 18% PEG 8000, and 0.1 M Bis-Tris propane (pH 9.0). STAU-229 Fab was crystallized in a solution containing 0.1 M ammonium acetate, 0.1 M Bis-Tris (pH 5.5), and 17% PEG 10000. Images were indexed and scaled with X-ray Detector software (41), and molecular replacement was performed in Phaser (42), followed by manual refinement using subsequent rounds of COOT (43) and Phenix (42).

### Constraint-guided docking using Rosetta.

Rigid-body docking was performed individually for both mAb-IsdB pairs by generating a starting ensemble, running the docking protocol with the applied constraints and filtering by overall energy, binding energy as calculated with the Rosetta InterfaceAnalyzer, and the ability to satisfy the constraints derived from HDX data (25, 26, 44). After a first round of docking, the best 500 models were selected by binding energy (interface energy) and underwent the protocol again, resulting in improved binding and energy scores. In total, 6,000 models were generated, and an ensemble of 25 best-scoring models by binding energy, which fulfilled the constraints derived from HDX data, were selected.

### Statistical analysis.

All data were analyzed in Prism v 7.0 (GraphPad Software Inc.). Individual statistical analyses are described in the legends of the figures.

### Data availability.

Crystal structures have been deposited in the PDB under accession numbers 6P9H for Fab STAU-281 in complex with the NEAT2 domain of IsdB, 6P9I for Fab STAU-399, and 6P9J for FAb STAU-229.

10.1128/mBio.02473-19.1 TEXT S1 Supplemental methods, including human mAb and Fab production, antibody variable gene sequence analysis, bioinformatics and processing of next-generation sequencing (NGS) data, and phylogenetic analysis. Download Text S1, PDF file, 0.03 MB. Copyright © 2019 Bennett et al. 2019 Bennett et al. This content is distributed under the terms of the Creative Commons Attribution 4.0 International license.
