# Supplementary material for: Human VH1-69 Gene-Encoded Human Monoclonal Antibodies against Staphylococcus aureus IsdB Use at Least Three Distinct Modes of Binding To Inhibit Bacterial Growth and Pathogenesis
Source: mBio. 2019 Oct 22;10(5):e02473-19. doi: 10.1128/mBio.02473-19 (PMC6805997; doi:10.1128/mBio.02473-19)
Supplement: TABLE S1 [file mBio.02473-19-st001.pdf]

Supplemental Table 1. Data collection and refinement statistics for the crystals

Data collection			
Crystal	STAU-281/Neat2	STAU-399	STAU-229
PDB ID	6P9H	6P9I	6P9J
Wave Length (Å)	0.97872	0.97856	0.97872
Space group	$P2_1$	$P2_1$	$P2_1$
Unit cell dimensions			
a, b, c (Å)	72.6, 115.3, 88.3	49.6, 99.9, 93.9	57.8, 66.0, 112.3
α, β, γ	90.0, 100.1, 90.0	90.0, 96.3, 90.0	90.0, 96.4, 90.0
Resolution (Å)	48.04 – 3.00	49.29 – 2.40	48.92 – 2.20
Unique reflections	28660 (4141)	35657 (5197)	43034 (6144)
Redundancy	3.9 (3.8)	3.8 (3.9)	3.8 (3.8)
Completeness (%)	99.8 (98.9)	100.0 (100.0)	99.7 (98.0)
R_{merge} (%)	6.9 (31.6)	8.7 (58.1)	8.1 (78.8)
I/σ(I)	15.2 (3.8)	12.8 (2.4)	10.9 (1.8)
Refinement statitics			
R_{factor}	20.64	18.50	21.81
R_{free}	22.50	24.34	26.70
R.m.s.d. (bond) (Å)	0.0020	0.0016	0.0048
R.m.s.d. (angle) (deg)	0.519	0.482	0.722
Ramachandran plot			
Favored (%)	96.99	96.86	97.43
Allowed (%)	2.82	3.14	2.57
Outliers (%)	0.19	0.00	0.00

$R_{merge} = \Sigma\ \Sigma\ |I_{hkl} - I_{hkl(j)}|/\Sigma\ I_{hkl}$, where $I_{hkl(j)}$ is the observed intensity and I_{hkl} is the final average intensity. $R_{work} = \Sigma\ ||Fobs| - |Fcalc|/\Sigma\ |Fobs|$ and $R_{free} = \Sigma\ ||Fobs| - |Fcalc||/\Sigma\ |Fobs|$, where R_{free} and R_{work} are calculated using a randomly selected test set of 5% of the data and all reflections excluding the 5% test set, respectively. Numbers in parentheses are for the highest resolution shell.
