# Supplementary material for: Human VH1-69 Gene-Encoded Human Monoclonal Antibodies against Staphylococcus aureus IsdB Use at Least Three Distinct Modes of Binding To Inhibit Bacterial Growth and Pathogenesis
Source: mBio. 2019 Oct 22;10(5):e02473-19. doi: 10.1128/mBio.02473-19 (PMC6805997; doi:10.1128/mBio.02473-19)
Supplement: FIG S2 [file mBio.02473-19-sf002.pdf]

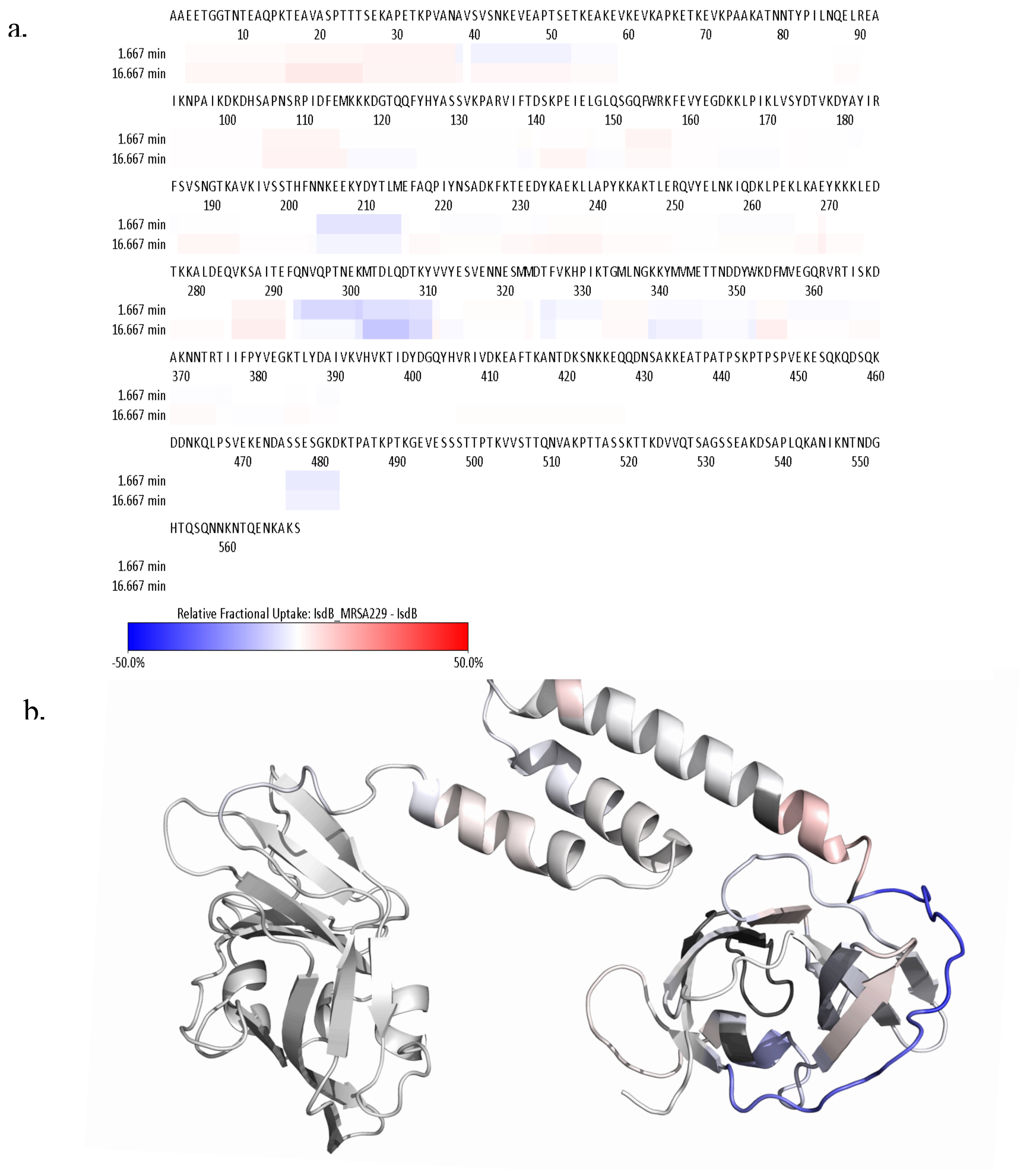


**Supplemental Figure 2. HDX-MS of IsdB-NEAT with STAU-229**

(a) Heat map showing the difference in relative deuterium-uptake of IsdB in complex with STAU-229 Fab compared to unbound IsdB. The sequence of IsdB is shown and overlapping peptides are shown below. Shielded amino acids (blue) indicate potential binding-sites and are boxed. (b) The structure of crystalized IsdB is shown. Coloring represents the difference in deuterium-uptake in complex to that in the solitary IsdB from HDX. Scale ranges from -20 (blue) to +20 (red), black represents not observed regions.
