# Supplementary material for: Human VH1-69 Gene-Encoded Human Monoclonal Antibodies against Staphylococcus aureus IsdB Use at Least Three Distinct Modes of Binding To Inhibit Bacterial Growth and Pathogenesis
Source: mBio. 2019 Oct 22;10(5):e02473-19. doi: 10.1128/mBio.02473-19 (PMC6805997; doi:10.1128/mBio.02473-19)
Supplement: FIG S3 [file mBio.02473-19-sf003.pdf]

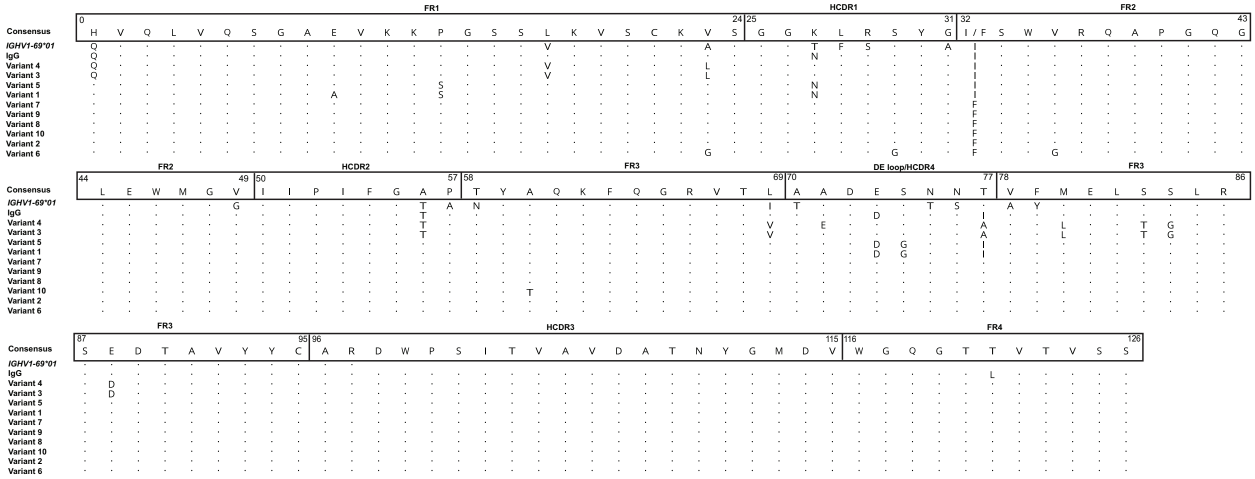

Supplemental Figure 3. ***Sequence alignment of STAU-399 and clonal variants.*** A global alignment using Geneious was performed with the germline *IGHV1-69*01* sequence, hybridoma IgG, and all clonal variants. Each HCDR and framework (FR) region is denoted with a consensus sequence shown. Point mutants that differ from the consensus are shown as individual amino acids, whereas conserved residues are represented as dots.
