# Supplementary material for: Human VH1-69 Gene-Encoded Human Monoclonal Antibodies against Staphylococcus aureus IsdB Use at Least Three Distinct Modes of Binding To Inhibit Bacterial Growth and Pathogenesis
Source: mBio. 2019 Oct 22;10(5):e02473-19. doi: 10.1128/mBio.02473-19 (PMC6805997; doi:10.1128/mBio.02473-19)
Supplement: FIG S6 [file mBio.02473-19-sf006.pdf]

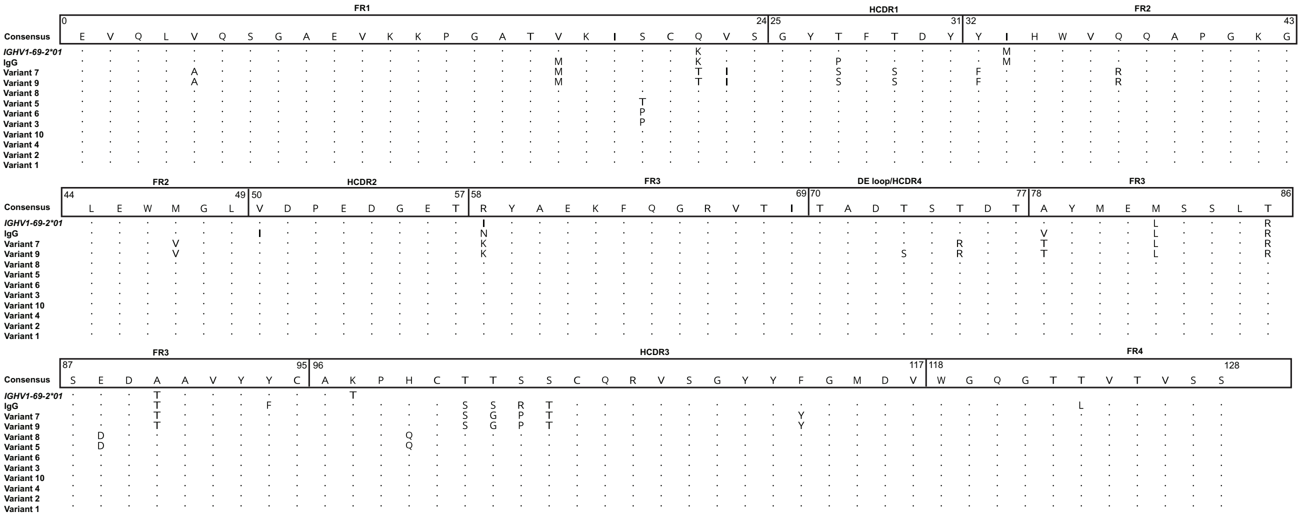

Supplemental Figure 6. ***Sequence alignment of STAU-229 and clonal variants*****.** A global alignment using Geneious was performed with the germline *IGHV1-69-2*01* gene sequence, the sequence from the hybridoma encoding the original IgG, and all STAU-229 clonal variants. Each HCDR and FR region is denoted, with a consensus sequence indicated. Point mutants that differ from the consensus are shown as individual amino acids whereas conserved residues are represented as dots.
