# Supplementary material for: Human VH1-69 Gene-Encoded Human Monoclonal Antibodies against Staphylococcus aureus IsdB Use at Least Three Distinct Modes of Binding To Inhibit Bacterial Growth and Pathogenesis
Source: mBio. 2019 Oct 22;10(5):e02473-19. doi: 10.1128/mBio.02473-19 (PMC6805997; doi:10.1128/mBio.02473-19)
Supplement: FIG S4 [file mBio.02473-19-sf004.pdf]

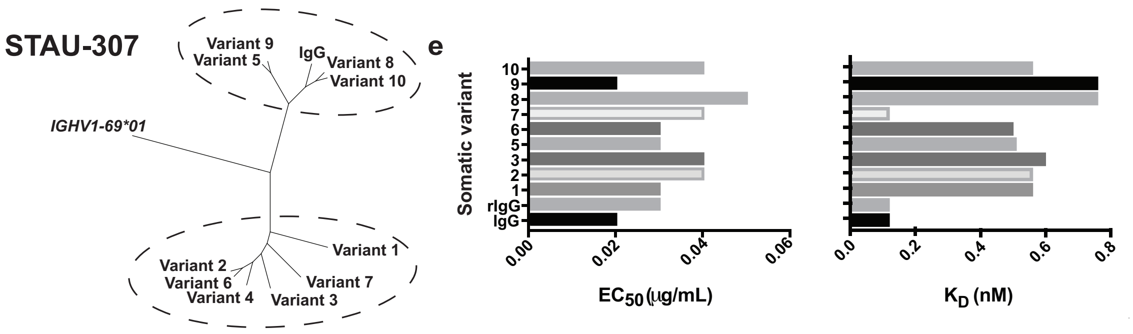

Supplemental Figure 4. ***Neighbor joining tree analysis and binding kinetics of STAU-307 and clonal variants.*** An additional *IGHV1-69*01* antibody (which binds site 2), STAU-307, was found to have clonal variants within the donor sample. Ten sequences of the STAU-307 clonotype (encoded by V_H*1-69*01/*J_H*4*02*) were identified, with two of these sequences being identical. Siblings were divided into two main clusters based on the phylogenetic analysis. Cluster 1 contains the original IgG and variants 5, 8-10, whereas cluster 2 contains variants 1-4, 6-7. Interestingly, the main differences between these two clusters occurs in the DE loop (Supplemental Figure 2), where three different runs of mutations occurs. All variants included in clusters 1 and 2 have mutations in their HCDR2 that differ from the germline *IGHV1-69*01* sequence. This finding is striking as a conserved HCDR2 can have important implications for blocking the heme pocket of NEAT2 as Figure 1 shows, however, none of the STAU-307 variants maintain 100% identity with the germline sequence. Instead, all 10 variants and the sequence from the hybridoma IgG have the mutations I51V and I53V. Although both isoleucine and valine are branched hydrophobic amino acids, mutating between these residues has been shown to alter function in previous studies (Yuan, X., Yin, P., Hao, Q., Yan, C., et al., 2010. Single amino acid alteration between valine and isoleucine determines the distinct pyrabactin selectivity by PYL1 and PYL2. J Biol Chem, 285, 28953-8.10.1074/jbc.M110.160192, and, Brosnan, J. T. & Brosnan, M. E. 2006. Branched-chain amino acids: enzyme and substrate regulation. J Nutr, 136, 207S-11S.10.1093/jn/136.1.207S). Despite the presence of these point mutations, these variants had largely the same ability to bind IsdB when tested by ELISA (Figure 4E). Variant 1, however, did have a noticeably worse K_D compared with the other variants tested. This difference may be attributed to the Y107D mutation found in the HCDR3 that is unique to this sequence.
