# Supplementary material for: Human VH1-69 Gene-Encoded Human Monoclonal Antibodies against Staphylococcus aureus IsdB Use at Least Three Distinct Modes of Binding To Inhibit Bacterial Growth and Pathogenesis
Source: mBio. 2019 Oct 22;10(5):e02473-19. doi: 10.1128/mBio.02473-19 (PMC6805997; doi:10.1128/mBio.02473-19)
Supplement: TEXT S1 [file mBio.02473-19-s0001.pdf]

Human mAb and Fab production

Fab fragments were expressed recombinantly by cloning the cDNA encoding the heavy and light chain variable gene regions into IgG expression vectors and transforming them into *E. coli*. MAb protein was produced by transient co-transfection of the Fab heavy chain and light chain DNA into either ExpiCHO or EXPI293F cells (ThermoFisher Scientific) following the manufacturer's protocol. Recombinant Fab was purified from centrifuged, 0.45 µM filtered supernatant using an Anti-CH1 column (GE Healthcare).

Antibody variable gene sequence analysis

Total RNA was isolated from approximately 15 million PBMCs using the Qiagen RNeasy Mini Kit (Qiagen, CA). Purified total RNA samples were processed at AbHelix, LLC (www.abhelix.com, South Plainfield, NJ, USA). Briefly, RNA samples were reversed transcribed using the oligo $d(T)_{18}$ in 3-5 µg per 20 µL reaction (SuperScript IV Reverse Transcriptase, ThermoFisher, CA). Multiple reactions of reverse transcription were combined and purified using magnetic beads. The purified RT products were divided evenly for the first round of PCR amplification specific to human IgG, IgK, IgL, IgM and IgA. The 5′ multiplex PCR primers were designed within the leader sequences for each productive V-gene and the 3′ primers within the constant regions, but in close approximation to the J-C junctions. The resulting 1st round PCR products were purified with magnetic beads and subjected to a second round of PCR amplification to add Illumina index and adapter sequences. The resulting PCR products were purified with magnetic beads and

pooled for sequencing with a PE250 protocol on an Illumina MiSeq sequencer. Phusion High-Fidelity DNA Polymerase (ThermoFisher, CA) was used in all PCR amplification reactions and care was taken to minimize the number of cycles to achieve adequate amplification. For IgG sequencing reads, subclass-specific sequences in the beginning of the constant regions were used to identify IgG1, IgG2, IgG3, or IgG4 subclasses.

Bioinformatics and processing of next-generation sequencing (NGS)

The bioinformatics processing of all NGS data was done using our PyIR sequence processing pipeline (https://github.com/crowelab/PyIR) with sample and data management performed using our in-house proprietary laboratory information management system. We used BLAST[52] and searched through the processed NGS data using the heavy chain V3J clonotypes belonging to monoclonal *IGVH1-69* hybridoma sequences. A BLAST database was constructed using all the unique V3J clonotypes derived from the NGS data (data from all isotypes were pooled together). We set the sequence identity threshold to 70% and the sequence alignment coverage to 95%. The BLAST parameters were set using the following values: word size=3, gap open and extension parameters were to 7 and 2 respectively.

Phylogenetic analysis

Neighbor-joining tree analysis was performed using the Geneious 10 software. Trees were rooted using the germline sequence. The trees branches were transformed proportionally and are in increasing order.
